# Bioinformatics analysis to investigate the potential relationship between mitochondrial structure and function-related genes and the immune microenvironment in atherosclerosis

**DOI:** 10.3389/fcvm.2025.1526151

**Published:** 2025-05-13

**Authors:** Hanning Yang, Yue Sun, Shumin Li, Yueyue Tang, Yuxue Wang, Yunyan Li, Yongping Lu

**Affiliations:** Department of Ultrasound, The Affiliated Hospital of Yunnan University, Kunming, Yunnan, China

**Keywords:** atherosclerosis, mitochondria, immune microenvironment, inflammation, macrophage, single-cell analysis

## Abstract

**Objective:**

This study aims to elucidate the interactions between genes associated with mitochondrial structure and function and the immune microenvironment in atherosclerosis.

**Methods:**

Differentially expressed mitochondria-related genes (DE-MRGs) were identified through the analysis of two gene expression datasets, GSE100927 and GSE159677, in conjunction with a list of mitochondria-related genes sourced from the MitoCarta3.0 database. The immune profile of infiltrating immune cells in atherosclerotic carotid artery (CA) patients compared to controls (CTLs) was assessed using CIBERSORT. Potential target genes were screened based on Spearman correlation analysis between specific DE-MRGs and differentially expressed immune cells. Furthermore, the correlation between characterized DE-MRGs and immune cells in AS was examined at the single-cell level, and the expression of key genes was validated *in vitro*.

**Results:**

Our study identified a robust association between four key genes—C15orf48, UCP2, PPIF, and MGST1—among 15 DE-MRGs, and immune macrophage polarization. These genes exhibited alterations corresponding to the degree of macrophage differentiation in AS. Additionally, Gene Set Enrichment Analysis (GSEA) revealed that C15orf48, UCP2, PPIF, and MGST1 modulate multiple immune pathways within the body. The mRNA expression levels of these four key genes in AS were confirmed via quantitative real-time PCR (qRT-PCR), with results aligning with bioinformatics predictions. Compared to the control group, the expression levels of C15orf48, UCP2, and PPIF were significantly elevated in AS macrophages, whereas MGST1 expression was notably reduced in AS macrophages. Consequently, these mitochondria-related genes—C15orf48, UCP2, PPIF, and MGST1—may influence the immune microenvironment in AS by modulating macrophage differentiation.

**Conclusion:**

C15orf48, UCP2, PPIF, and MGST1 may serve as potential therapeutic targets for enhancing the atherosclerotic immune microenvironment in future interventions.

## 1 Introduction

AS is a chronic arterial disease characterized by the formation of plaques on the inner walls of arteries, leading to the narrowing and hardening of blood vessels (1). It is a major contributor to coronary artery disease, including acute myocardial infarction (AMI), as well as ischemic vascular diseases such as transient ischemic attack (TIA) and cerebral infarction (2). Consequently, AS is associated with cardiovascular-related mortality worldwide (2, 3). Despite the relatively low mortality rates from AS in many countries, the conditions induced by AS—such as coronary heart disease (CHD), ischemic stroke, and peripheral arterial disease (PAD)—significantly contribute to the economic and health burdens faced by patients and healthcare systems (4). While lipid-lowering drugs have proven effective in preventing and treating AS over the past few decades, the global incidence of AS has continued to rise from 1990 to 2019 (5). These findings underscore the urgent need for more sensitive and accurate diagnostic and therapeutic targets to improve the diagnosis and treatment of AS.

AS is fundamentally an inflammatory disease characterized by dysregulated immune cell activity (6). The infiltration of immune cells within the vascular wall is closely linked to both the stability and progression of AS (7). Emerging evidence indicates that the extent of immune cell infiltration and the presence of proinflammatory factors within the atherosclerotic immune microenvironment (AIM) are critical determinants of the development of AS (8). A diverse array of immune cells—including T cells, B cells, natural killer (NK) and NKT cells, macrophages, monocytes, dendritic cells (DCs), neutrophils, and mast cells—participate in various stages of the disease and its associated complications (9). In the context of AS, cytokines play pivotal roles by activating immune cells, modulating intercellular signaling, and fostering a local inflammatory milieu that promotes plaque formation (10). Consequently, reducing immune responses has emerged as a promising therapeutic strategy for the management of AS. A 2017 study revealed that suppressing the inflammatory response in patients with AS reduced their relative risk of atherosclerotic cardiovascular events by 15%, with significant reductions in blood hsCRP and IL-6 (11). Furthermore, additional studies have demonstrated that inhibiting the accumulation of immune cells within the arterial intima and attenuating the activation of vascular inflammatory pathways can significantly reduce the incidence of cardiovascular complications associated with AS (12). In summary, the evidence underscores the critical role of immune cell infiltration and proinflammatory factors in the pathogenesis of AS. Given the current research landscape, further exploration of the interplay between AS and immune responses represents a valuable avenue for future investigations and therapeutic interventions.

Mitochondria are essential organelles that play a critical role in maintaining cellular energy metabolism and homeostasis (13). Recent studies have demonstrated that mitochondrial microRNAs can influence the progression of AS by modulating ATP and reactive oxygen species (ROS) production. During the pathological progression of AS, the ultrastructures of mitochondrial networks become disrupted, leading to alterations such as matrix swelling, crista loss, and mitochondrial membrane rupture. These changes result in increased ROS production, damage to mitochondrial DNA (mtDNA), reduced mitochondrial respiration, and impaired lipid metabolism within cells (14, 15). The accumulation of excess lipids has been shown to interact with immune cells, triggering a cascade of inflammatory responses that culminate in the formation of atherosclerotic plaques. Importantly, research indicates that mitochondrial dysfunction can lead to decreased immune cell activity, increased cell death, and inappropriate activation of inflammatory responses, thereby creating a microenvironment conducive to the development of AS (16). Numerous pharmacological agents aimed at preventing AS by enhancing mitochondrial function have been extensively investigated (17). These include inhibitors of mitochondrial fission proteins, fusion-promoting agents, and antioxidants such as MitoQ, vitamin E, and curcumin (18, 19). However, the interplay between mitochondrial genes and the immune microenvironment in the context of AS remains underexplored.

Given the pivotal role of mitochondria in the pathogenesis of AS, this study aimed to investigate the involvement of mitochondria-related genes in immune processes through bioinformatics analysis. Our objective was to elucidate the interactions between mitochondrial structure- and function-related genes and the immune microenvironment in AS, thereby providing new insights into the potential mechanisms underlying AS and identifying novel targets for therapeutic intervention.

## 2 Materials and methods

### 2.1 Data sources

The datasets GSE159677 and GSE100927 were retrieved from the Gene Expression Omnibus (GEO) database (GSE159677:https://www.ncbi.nlm.nih.gov/geo/query/acc.cgi?acc=GSE159677; GSE100927:https://www.ncbi.nlm.nih.gov/geo/query/acc.cgi?acc=GSE100927). GSE159677: This dataset comprises single-cell sequencing data, including 3 normal samples and 3 samples from patients with carotid artery atherosclerosis. It is utilized for single-cell correlation analysis of the carotid artery. GSE100927: This dataset includes 29 samples from CA and 12 CTL. The GSE159677 dataset was generated via the Illumina NextSeq 500 platform (*Homo sapiens*), whereas the GSE100927 dataset was obtained via the Agilent-039494 SurePrint G3 Human GE v2 8  ×  60K Microarray (Probe Name version) platform. Mitochondria-related genes were sourced from the Human MitoCarta3 database (https://personal.broadinstitute.org/scalvo/MitoCarta3.0/human.mitocarta3.0.html), resulting in a total of 1,136 mitochondrial-related genes (MRGs) for subsequent analysis.

### 2.2 Single-cell data analysis

In this study, single-cell RNA sequencing data were processed and analyzed using the R package Seurat (version 5.1.0), which facilitated filtering, quality control, normalization, dimensionality reduction, and clustering. Initially, the CreateSeuratObject function was employed to filter the data, retaining genes expressed in at least 10 cells and cells with more than 200 detected genes (parameters: min.cells = 10, min.features = 200). Subsequently, the PercentageFeatureSet function was utilized to calculate the proportion of mitochondrial genes, and cells with mitochondrial gene percentages below 10% were retained for further analysis. The data normalization was performed using the NormalizeData function, followed by feature selection using the FindVariableFeatures function to identify genes with high variability across cells. Principal component analysis (PCA) was then applied to reduce the dimensionality of the data, and inflection points were plotted to determine the optimal number of dimensions for downstream analysis. Based on the inflection point, the first 30 principal components were selected for clustering. Positive marker genes for each cluster were identified using the FindAllMarkers function (parameters: min.pct = 0.6, only.pos = TRUE, logfc.threshold = 0.5), with the Wilcoxon rank-sum test employed for differential expression analysis (one cluster vs. all others). Cell type annotation was performed by identifying marker genes for each subpopulation and comparing them with reference marker genes from the CellMarker database and other published resources. Manual annotation was then used to assign cell types to each cluster, and the proportions of each cell subpopulation were calculated. Additionally, macrophage (Macro) data were extracted for further analysis. Differential expression analysis between CA and CTL groups within macrophages was conducted using the differentialGeneTest function, with genes meeting the threshold of FDR ≤0.00001 selected for subsequent investigation. To construct cell trajectories, dimensionality reduction and cell ordering were performed using the DDRTree method, enabling the visualization of developmental pathways and cellular transitions.

### 2.3 Identification of the differentially expressed genes

Differentially expressed genes were identified via the limma package (version 3.60.3) in R. The screening criteria applied were |logFoldChange| >1 and adjusted *p*-value (padj) <0.05.

### 2.4 Gene set variation analysis (GSVA)

KEGG pathway scores were calculated via GSVA (version 1.52.3) on the basis of the KEGG gene set.

### 2.5 Gene set enrichment analysis (GSEA)

Using transcriptome data, CA samples were categorized into high- and low-expression groups on the basis of the expression levels of target genes for differential analysis, resulting in the calculation of the log2-fold change (log2FC). The log2FC values were then sorted from high to low, and this ranking was used to perform gene set enrichment analysis (GSEA) via the clusterProfiler package (version 4.12.0). This analysis aimed to explore the pathways associated with key genes and their functional roles. The results were filtered using the criteria of adjusted *p*-value (padj) <0.05 and |normalized enrichment score (NES)| >1. The top 10 results based on padj ranking were visualized via GSEA ridge diagrams generated with GseaVis (version 0.0.5).

### 2.6 Cell culture and treatment

Bone marrow-derived macrophages (BMDM) were cultured in RPMI-1640 medium (Cytiva), enriched with 10% fetal bovine serum (Gibco), 1% penicillin/streptomycin (Gibco), and 25 ng/ml macrophage colony-stimulating factor (eBioscience). The cells were incubated for seven days at 37°C to facilitate differentiation into mature macrophages, specifically the M0 phenotype. To establish the atherosclerosis model, the differentiated macrophages were treated with 0.5 mmol/ml palmitic acid (PA, Sigma-Aldrich) for 24 h. Control groups were treated with an equivalent volume of dimethyl sulfoxide (DMSO).

### 2.7 RNA extraction and real-time quantitative PCR (qPCR)

Total RNA was extracted from BMDM using TRIzol reagent (China). Complementary DNA (cDNA) was synthesized using a reverse transcription kit. Quantitative PCR (qPCR) was conducted with a SYBR Green real-time PCR premix kit (AG). All primers were synthesized by Beijing Kinko's Biotechnology Company (Supplementary Table S1). The 2^-ΔΔCt method was employed to compare and calculate the relative quantitative values, with GAPDH serving as the internal reference gene for qPCR.

### 2.8 Statistical analysis

Data were analyzed using R software (version 4.4.0, https://www.r-project.org/) and GraphPad Prism software. The Wilcoxon rank sum test was used to compare gene expression differences and immune cell infiltration differences between different groups. When *P*-value <0.05, it was considered statistically significant.

## 3 Results

### 3.1 Mitochondria-related gene screening in AS

**Figure 1:**
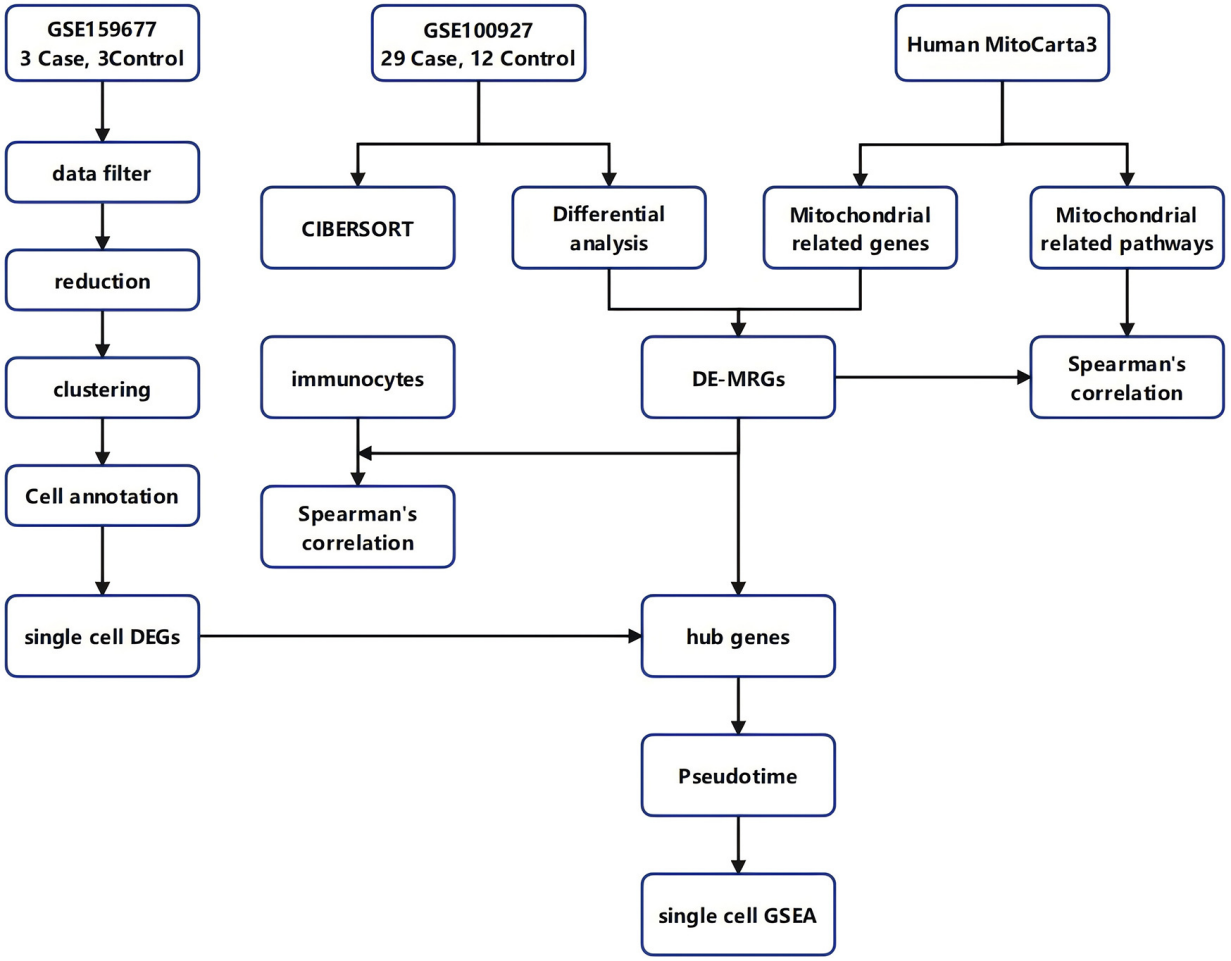
Workflow diagram. The flowchart of this study.

**Figure 2:**
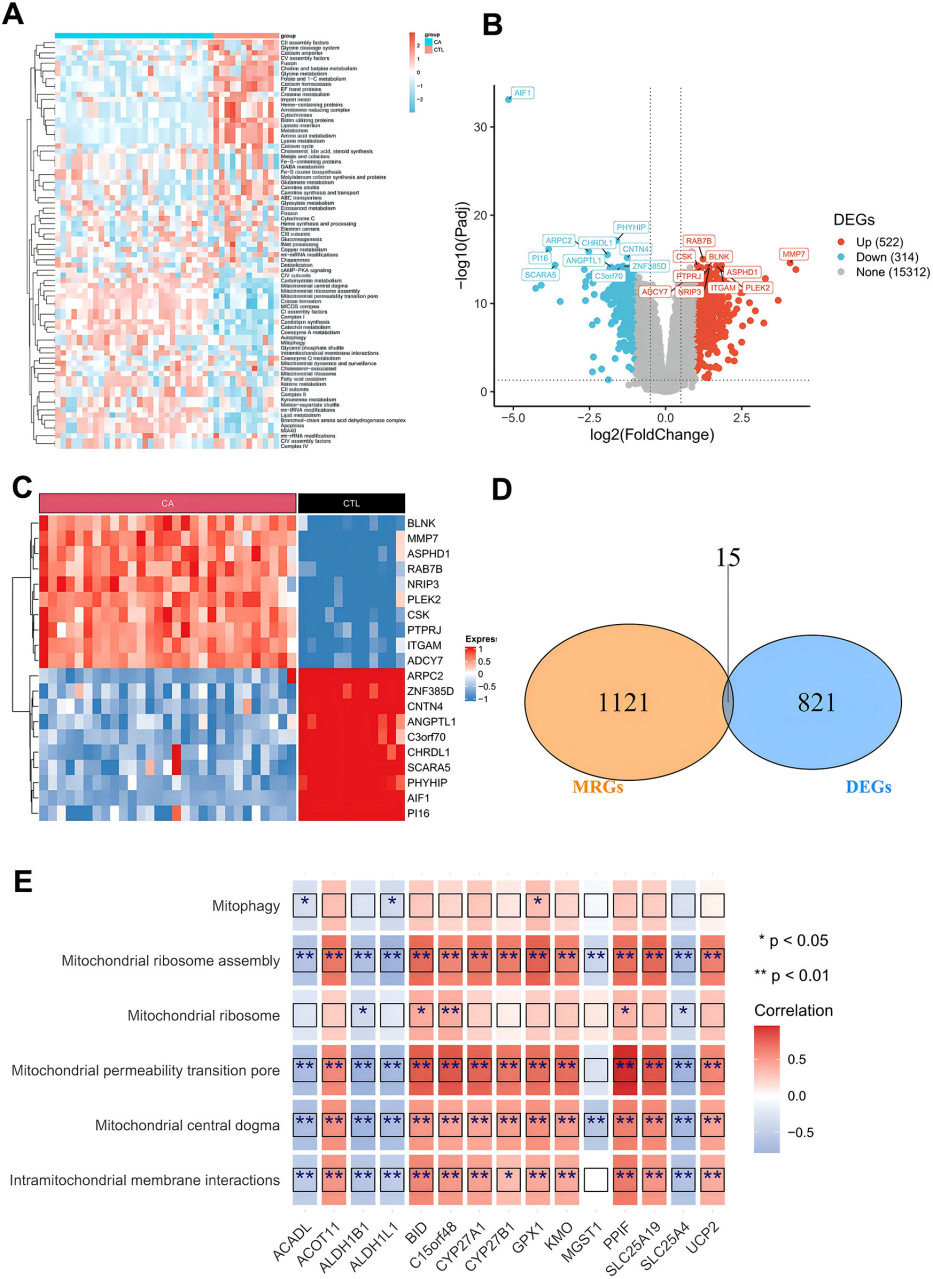
Screening of differentially expressed mitochondria-associated genes. **(A)** Differential mitochondria-associated pathways between CA/CTL samples; **(B)** volcano plot of differentially expressed genes between CA/CTL samples; **(C)** heatmap of differentially expressed genes between CA/CTL samples; **(D)** Wayne's plot of the intersection of MRGs and DEGs; and **(E)** heatmap of the correlation of DE-MRGs with mitochondrial pathways.

The workflow of our study is presented in Figure 1. We conducted target screening on the basis of 149 pathways related to mitochondrial structure and function obtained from the Human MitoCarta 3.0 database (Supplementary Table S2). Following pathway scoring calculations via gene set variation analysis (GSVA), we identified a total of 79 mitochondria-associated pathway scores that were significantly different between the CA and CTL groups (Supplementary Table S3, Figure 2A). We subsequently screened for differentially expressed genes (DEGs) between the CA and CTL groups via the GSE100927 database. Our analysis revealed a total of 4,081 DEGs in the CA samples compared with those in the CTL samples, with 2,213 genes upregulated and 1,868 genes downregulated (Figures 2B,C). We then intersected the MRGs identified from our screening with the DEGs, resulting in a total of 15 genes, which we designated DE-MRGs (Supplementary Table S4, Figure 2D). To further validate the accuracy of our screening results, we randomly selected one of the 15 DE-MRGs for Spearman correlation analysis with the mitochondria-associated pathways. The results demonstrated a strong correlation between the two (Figure 2E), confirming the reliability of our findings.

### 3.2 Correlation analysis of DE-MRGs with immune cell infiltration

**Figure 3:**
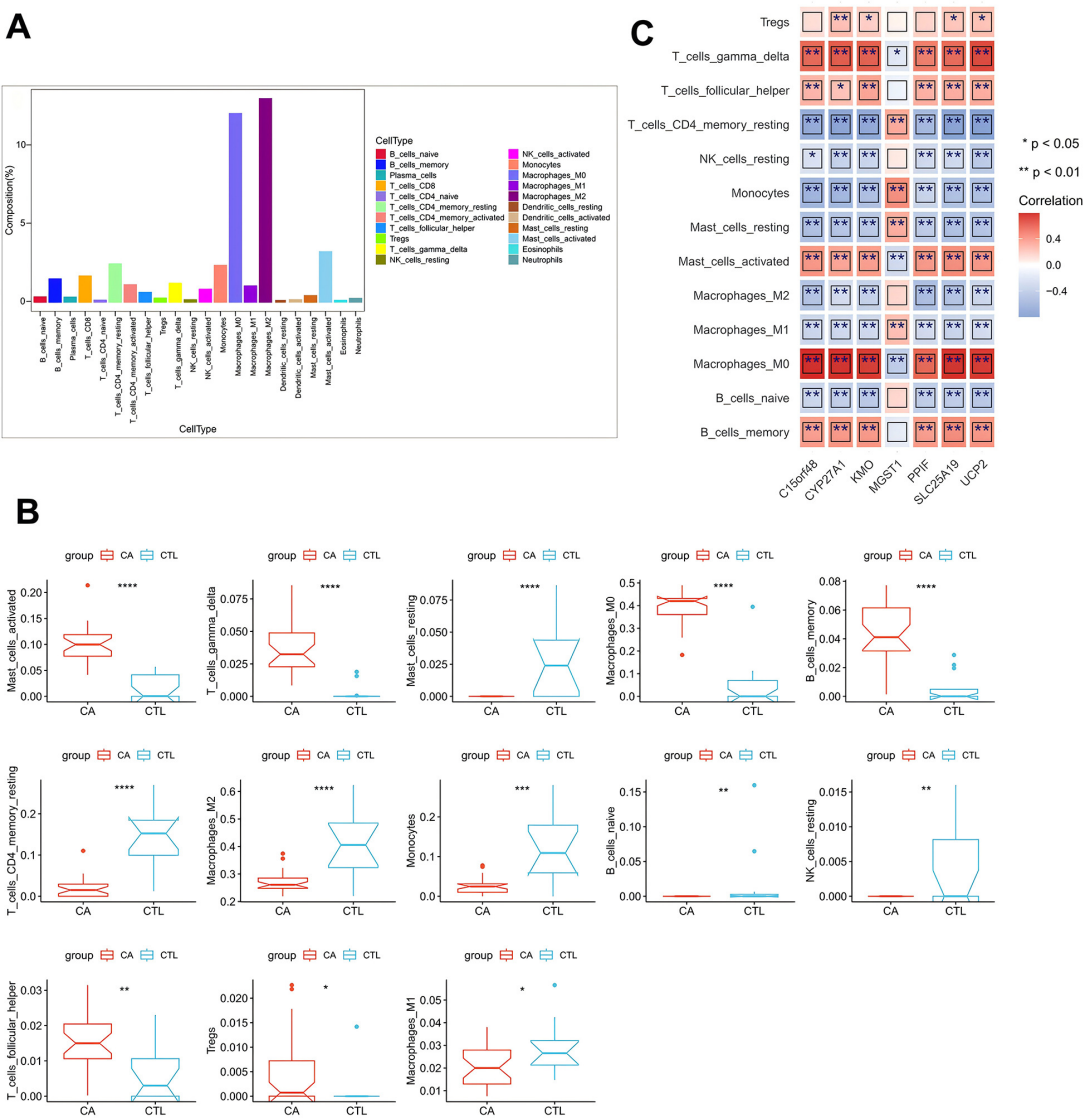
DE-MRG and immune cell correlation analysis. **(A)** Box line plot of immune cell proportions; **(B)** box line plot of immune cell proportion differences; **(C)** heatmap of correlations between key genes and differential immune cells. *represents *P* < 0.05, **represents *P* < 0.01, ***represents *P* < 0.001, ****represents *P* < 0.0001; red represents a positive correlation, and blue represents a negative correlation.

To assess the significance of DE-MRGs in AS immune microenvironment, we analyzed the correlations between the 15 DE-MRGs and immune cell infiltration as well as immune function. We first calculated the proportions of 22 immune cell types across all samples in the GSE100927 database via the CIBERSORT algorithm with IOBR (version 0.99.8). This allowed us to determine the proportion of each immune cell type in each sample. Box line plots were generated via the ggpubr package to illustrate the distribution of immune cell proportions across all samples. Our analysis revealed that Macrophages_M0 and Macrophages_M2 had the highest proportions in the CA group (Figure 3A). Next, we compared the immune cell proportions between the CTL and CA groups. The results revealed significant differences in the proportions of several immune cells, including activated mast cells, resting mast cells, M0 macrophages, memory B cells, M2 macrophages, monocytes, M1 macrophages, and 13 other immune cell types. Notably, the proportions of M1 and M2 macrophages were decreased in the CA group (Figure 3B). In addition, we conducted a Spearman correlation analysis between the 15 DE-MRGs and various immune cell types. The analysis revealed that DE-MRGs exhibited significant correlations with all 13 types of differential immune cells (Figure 2E). Notably, the strongest correlation was observed between DE-MRGs and macrophages. These findings preliminarily suggest that mitochondrial-related genes are significantly associated with macrophage immune infiltration in the context of AS.

### 3.3 Single-cell data analysis

**Figure 4:**
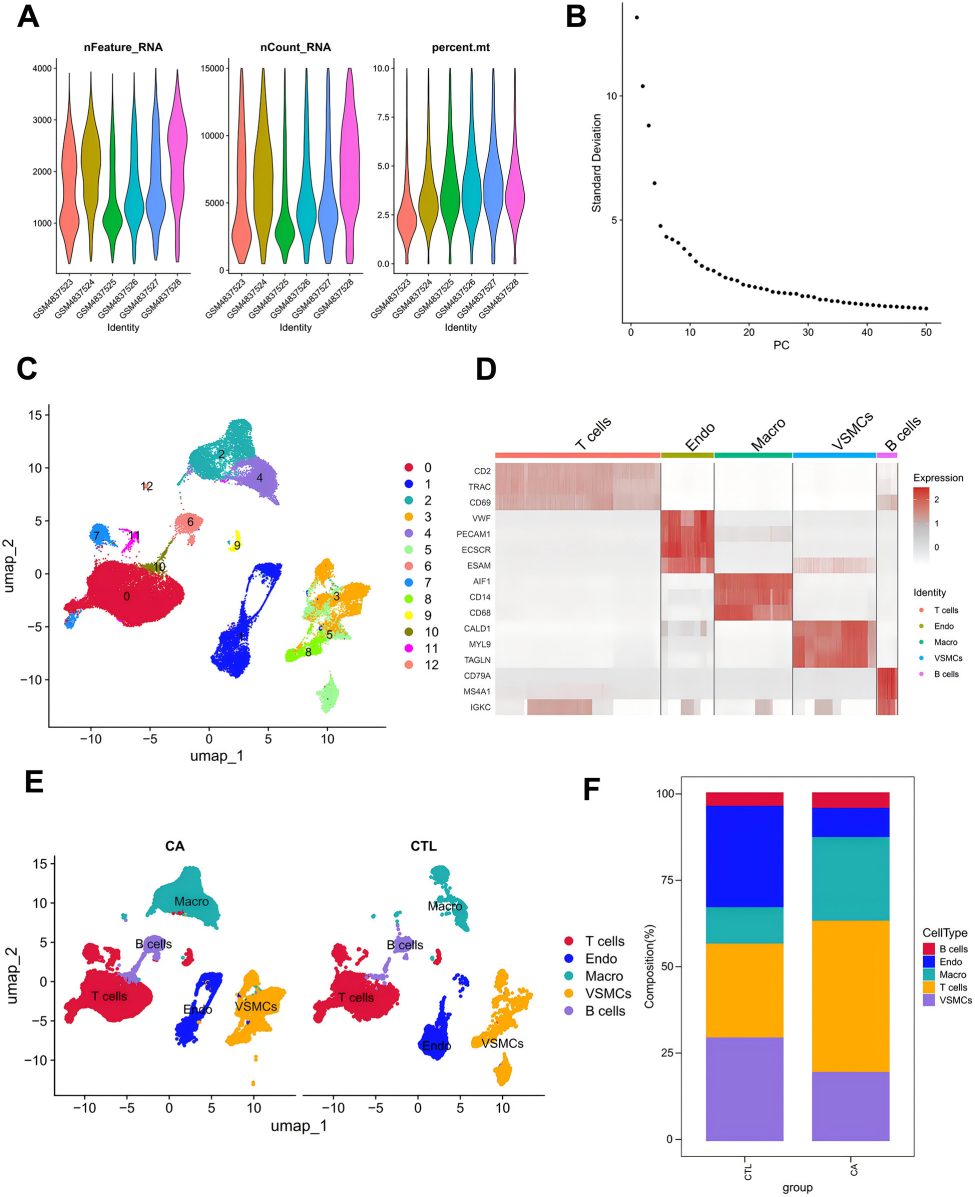
Single-cell data analysis. **(A)** Characteristic violin plots of single-cell sequencing data; **(B)** inflection point plots; **(C)** cell clustering-UMAP; **(D)** cell marker expression heatmap; **(E)** manually annotated cell subpopulations; and **(F)** histograms of the proportions of single-cell subpopulations.

To further elucidate the relationship between DE-MRGs and immune cells, we conducted validation using single-cell sequencing data. Following rigorous filtering and mitochondrial gene calculation, we successfully obtained a dataset comprising 43,065 cells and 19,162 genes (Figure 4A). The dataset was subsequently subjected to principal component analysis (PCA) for dimensionality reduction, and inflection plots were generated to identify the optimal dimensions for analysis. Based on this evaluation, we selected the top 30 principal components for subsequent clustering analysis (Figure 4B). The unsupervised clustering analysis revealed the presence of 12 distinct clusters (Figure 4C). We proceeded to identify the cell types within these clusters by determining the marker genes for each subpopulation. This involved comparing the identified cell genes with marker genes from the CellMarker database and relevant literature to accurately classify the cell subpopulations (Figure 4D). Through meticulous manual annotation, we identified five distinct cell subpopulations: macrophages, T cells, B cells, endothelial cells, and vascular smooth muscle cells (VSMCs) (Figure 3E). Quantitative analysis of the cell subpopulation proportions revealed a downregulation of endothelial cells and VSMCs in the CA group, whereas T cells and macrophages exhibited upregulated proportions (Figure 4F). The increased presence of T cells and macrophages underscores the significant association between DE-MRGs and immune cell infiltration in the context of AS.

### 3.4 Expression analysis of DE-MRGs in single cells

**Figure 5:**
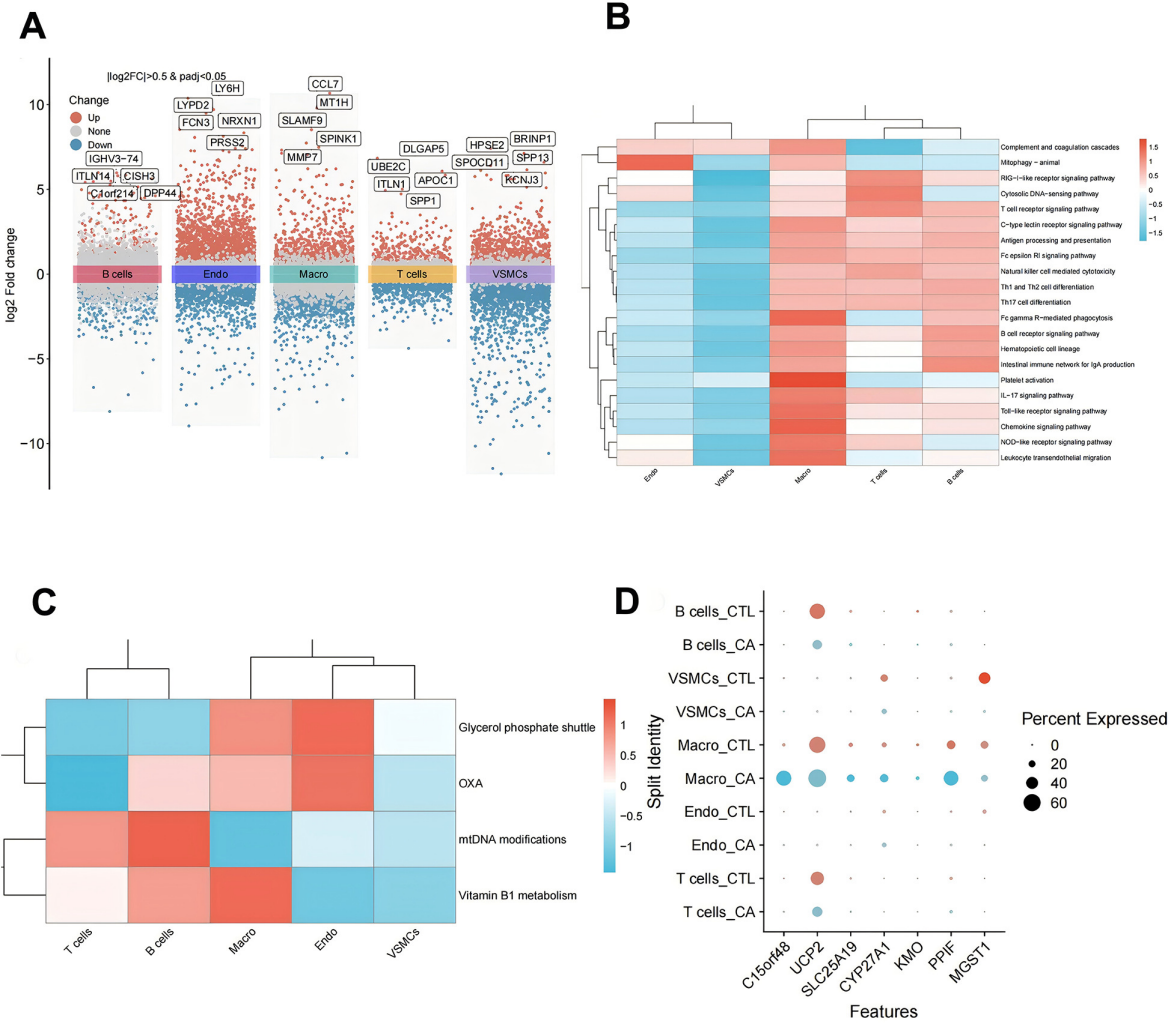
Expression analysis of DE-MRGs in single cells. **(A)** Single-cell differential gene volcano map; **(B)** single-cell subpopulation immune-related pathway scoring heatmap; **(C)** single-cell subpopulation mitochondrial pathway scoring heatmap; and **(D)** single-cell subpopulation key gene expression point map.

On the basis of the data obtained from the single-cell analysis described above, we identified the DEGs in the five cell subpopulations. The results revealed a total of 9,465 DEGs in macrophages, 13,525 DEGs in T cells, 4,219 DEGs in B cells, 9,119 DEGs in endothelial cells, and 2,561 DEGs in VSMCs (Figure 5A). We conducted an integrative analysis of the expression of differentially expressed DE-MRGs in both transcriptomic data and single-cell datasets. Our findings revealed that DE-MRGs exhibited similar differential expression patterns between the CA and CTL groups at the single-cell level. By focusing on DE-MRGs with consistent expression trends across both transcriptomic and single-cell analyses, we identified macrophages as the cellular subpopulation with the highest number of DE-MRGs compared to other subpopulations. Notably, the genes C15orf48, UCP2, SLC25A19, CYP27A1, KMO, and PPIF were significantly upregulated in macrophages, while the MGST1 gene was significantly downregulated in macrophages (Table 1). Further functional analysis of the differences among the five cellular subpopulations revealed that multiple immune-related pathways were more active in macrophages than in other cells. These pathways included platelet activation, Fc gamma R-mediated phagocytosis, and the chemokine signaling pathway (Figure 5B). Additionally, mitochondria-associated pathways were also more active in macrophages (Figure 5C). Taken together, these results underscore the significant role of macrophages, leading us to label the DE-MRGs as key genes in macrophages for subsequent analysis (Figure 5D). Finally, we identified four genes—C15orf48, UCP2, PPIF, and MGST1—that were more highly expressed in macrophages.

**Table 1:** Expression of DE-MRGs in the transcriptome and in single cells.

Gene	logFC	avg_log2FC	*P*-value	Cell
C15orf48	2.139	4.895	**5.119e-154**	Macro
C15orf48	2.139	4.579	9.736e-13	Endo
ALDH1B1	−1.105	1.513	3.222e-30	VSMCs
ALDH1B1	−1.105	1.126	0.0063	Macro
C15orf48	2.139	2.410	1.991e-07	T cells
ALDH1L1	−1.766	0.949	0.0006	VSMCs
UCP2	1.700	0.869	7.629e-05	T cells
UCP2	1.700	0.223	**5.901e-06**	Macro
SLC25A19	1.211	1.194	**9.058e-21**	Macro
BID	1.116	0.838	0.0006	VSMCs
CYP27A1	1.073	1.560	**7.8649e-22**	Macro
CYP27A1	1.073	−0.402	9.652e-19	VSMCs
CYP27A1	1.073	0.951	5.307e-10	Endo
KMO	1.048	0.580	**0.0006**	Macro
PPIF	1.029	1.189	**2.909e-44**	Macro
PPIF	1.029	0.457	0.0264	Endo
ACOT11	1.013	3.242	2.573e-10	Endo
MGST1	−1.071	−0.193	**0.0045**	Macro
MGST1	−1.071	−4.311	0	VSMCs
MGST1	−1.071	−1.947	2.573e-10	Endo
SLC25A4	−1.123	0.409	7.693e-10	VSMCs
SLC25A4	−1.123	0.548	8.951e-06	Endo
ALDH1B1	−1.105	−0.434	0.0096	Endo
UCP2	1.700	−0.246	7.092e-24	T cells
ACADL	−1.481	−0.547	2.152e-11	VSMCs
UCP2	1.700	−0.869	2.078e-19	B cells

Note: Genes marked in red are genes whose expression trends are consistent between the transcriptome and the single-cell analysis.

Bold value indicates *P*-value of DE-MRG gene in macrophages.

### 3.5 Pseudotemporal developmental trajectory analysis of macrophages in atherosclerosis

**Figure 6:**
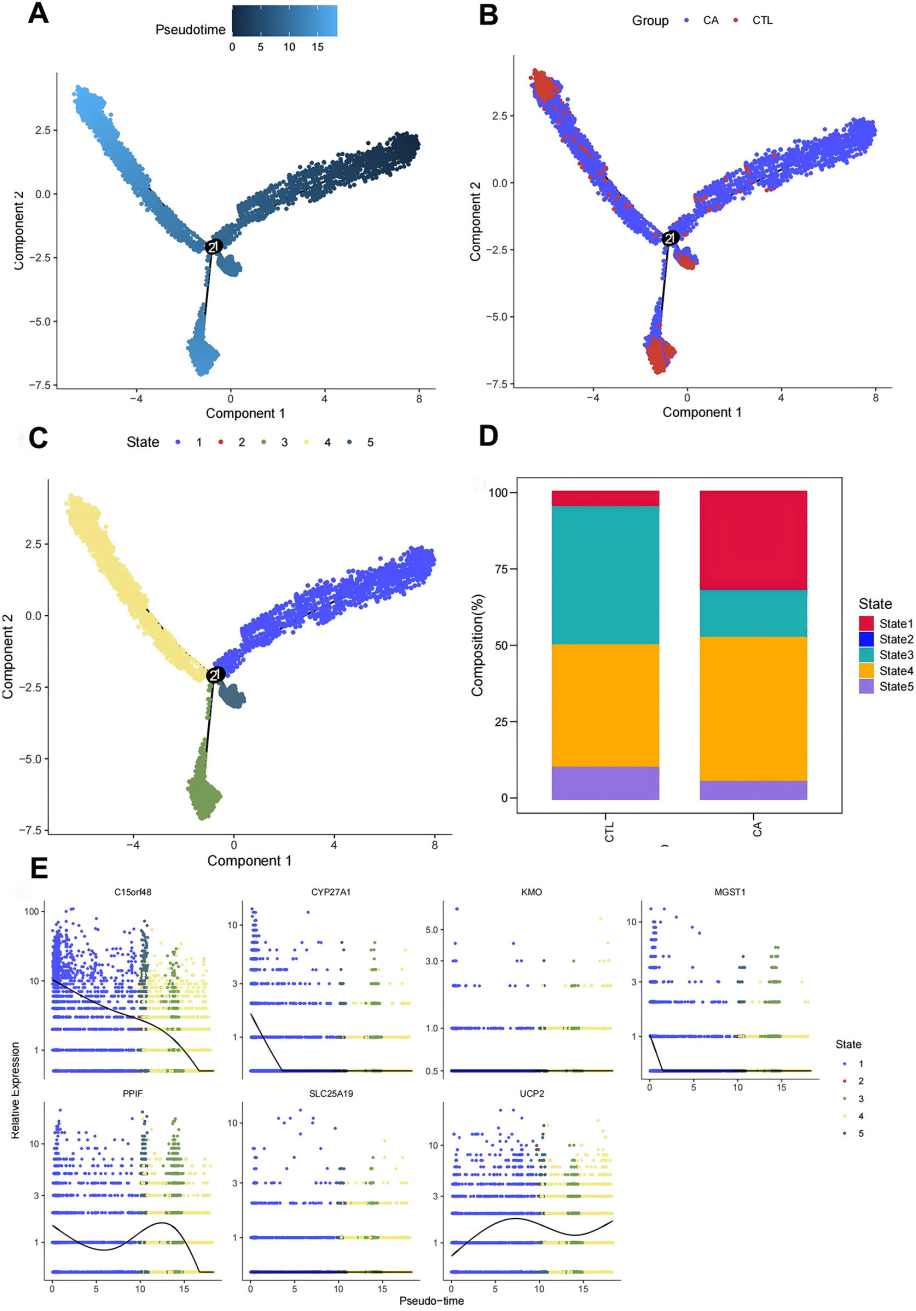
Macrofitting time series analysis. **(A–C)** Macromimetic time series analysis; **(D)** histogram of cell proportions in different branches; **(E)** scatter plot of key gene expression in different states.

To further understand the immunodynamics of macrophages in AS, we conducted a pseudotemporal developmental trajectory analysis of macrophages independently. By extracting macro data and utilizing the differentialGeneTest function, we identified genes that were differentially expressed between the CA and CTL groups. The results indicated a trend of differentiation in macrophages from left to right and from top to bottom, classifying them into five distinct states, with the highest degree of differentiation observed in State4, followed by State3 (Figures 6A‒C). We then quantified the ratio of macrophages in different states. The results revealed that the proportion of Macrophages in State 3 and State 4 was greatest in the CTL group, whereas fewer cells were found in State 1, State 2, and State 5. These findings suggest that most macrophages in the CTL group were able to differentiate and perform their functional functions. In contrast, in the CA group, while differentiation into State3 and State4 occurred, there was a notable increase in the proportion of Macrophages in State1 (which represents undifferentiated cells). These findings indicate that macrophage differentiation is inhibited following the onset of AS, which is consistent with the decrease in the proportions of M1 and M2 cells observed via transcriptome analysis (Figure 6D).

Finally, we examined the expression of DE-MRGs to determine their association with macrophage differentiation in AS. The assay results demonstrated that the DE-MRGs—C15orf48, UCP2, PPIF, and MGST1—were altered with the degree of differentiation. Thus, we hypothesized that these mitochondria-related genes could influence the immune microenvironment of AS by affecting macrophage differentiation (Figure 6E).

### 3.6 GSEA of key genes

**Figure 7:**
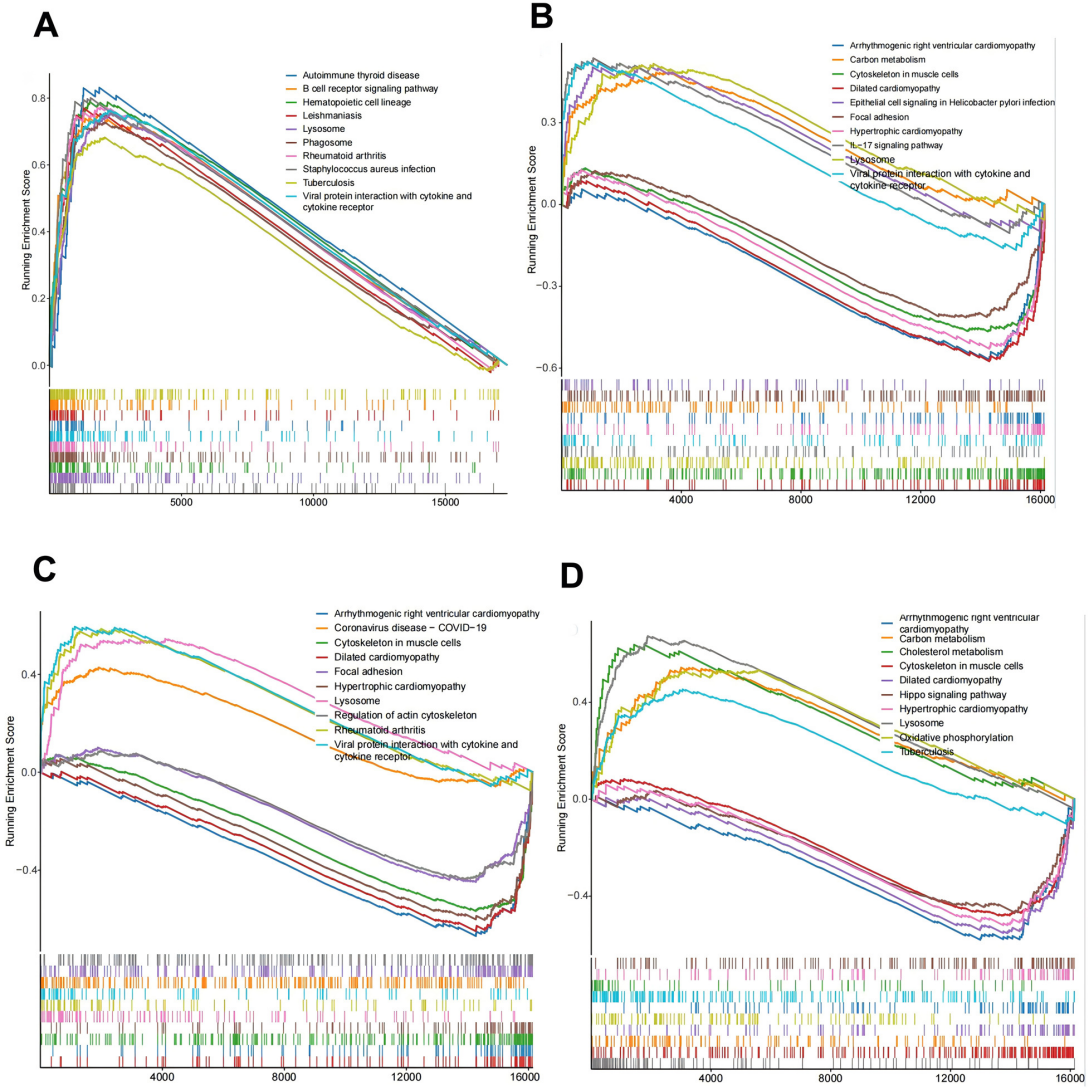
Key gene function prediction. **(A)** C15orf48-KEGG enrichment ridge map; **(B)** MGST1-KEGG enrichment ridge map; **(C)** PPIF-KEGG enrichment ridge map; **(D)** UCP2-KEGG enrichment ridge map.

To further explore the pathways and functions of the key genes, we performed single-gene GSEA of the target genes and visualized the top 10 GSEAs. The results revealed that the lysosome, phagosome, and B-cell receptor signaling pathways were significantly associated with C15orf48 (Figure 7A). The cytoskeleton in muscle cells, lysosomes, the IL-17 signaling pathway, carbon metabolism, focal adhesion, epithelial cell signaling in *Helicobacter pylori* infection, etc., were significantly associated with MGST1 (Figure 7B). The cytoskeleton in muscle cells, lysosomes, focal adhesion, regulation of the actin cytoskeleton, etc., were significantly correlated with PPIF (Figure 7C). Lysosomes, the cytoskeleton in muscle cells, carbon metabolism, oxidative phosphorylation, cholesterol metabolism, and the Hippo signaling pathway were significantly correlated with UCP2 (Figure 7D). Taken together, these results indicate that the screened DE-MRGs are involved in the regulation of multiple immune pathways, which further suggests that C15orf48, UCP2, PPIF, and MGST1 may be potential targets for improving the AS-free microenvironment.

### 3.7 Expression detection of key genes in AS macrophages

**Figure 8:**
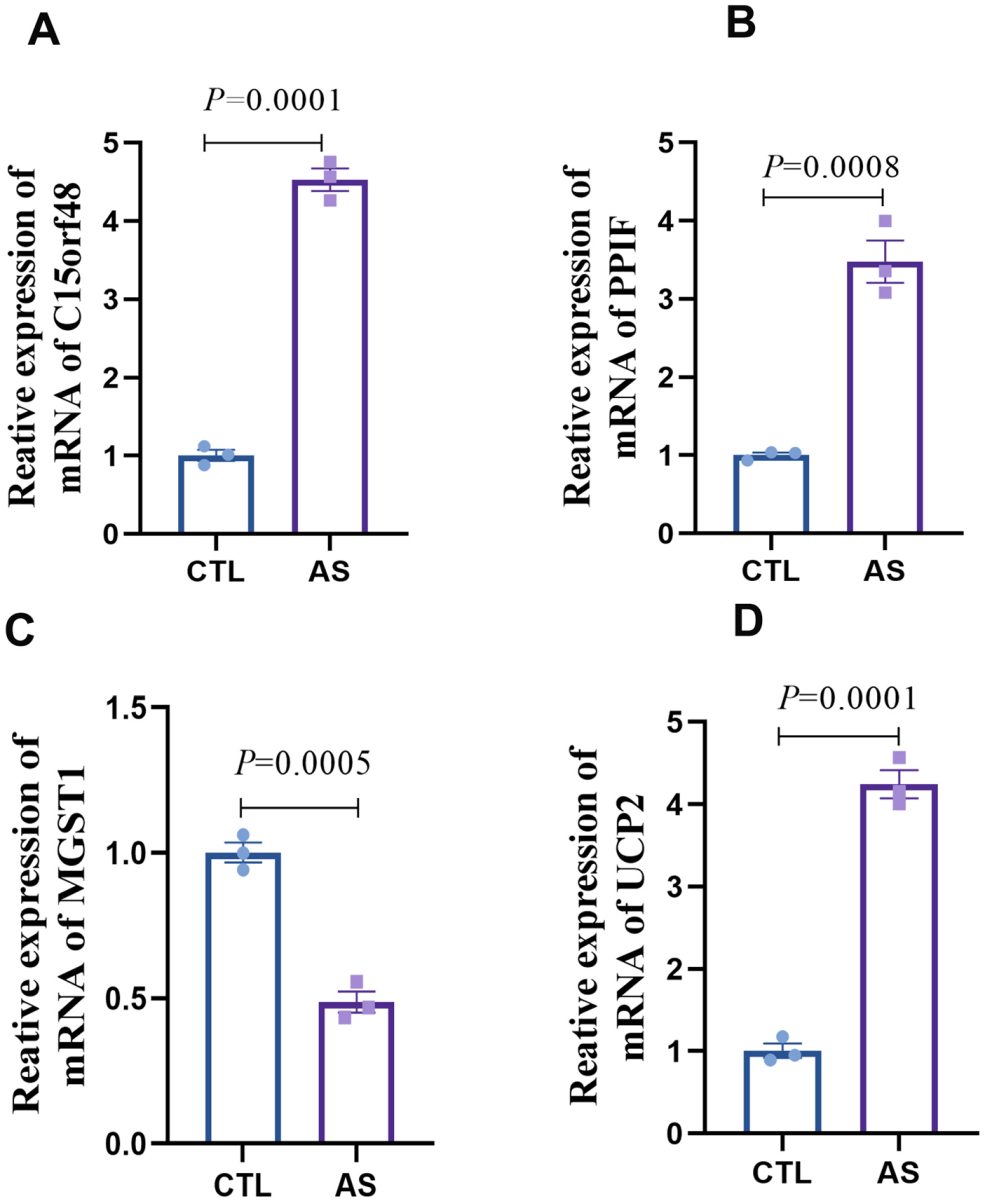
Expression detection of key genes in AS macrophages **(A)** statistical graph of C15orf48 mRNA expression level; **(B)** statistical graph of PPIF mRNA expression level; **(C)** statistical graph of MGST1 mRNA expression level; **(D)** statistical graph of UCP2 mRNA expression level.

Based on our analysis, we have identified that key mitochondrial genes—C15orf48, UCP2, PPIF, and MGST1—may play a crucial role in the development of AS by modulating the immune microenvironment through the somatic regulation of macrophage differentiation. To validate our findings, we developed an *in vitro* AS model using human macrophages and conducted transcriptomic analyses to examine the expression of these key genes in AS-associated macrophages. The results demonstrated differential expression of C15orf48, UCP2, PPIF, and MGST1 in the model group compared to the control group (Figures 8A–D). Specifically, the expression levels of C15orf48, PPIF, and UCP2 were significantly upregulated, while the expression level of MGST1 was significantly downregulated in the AS group. These validation results align with the differential expression levels of DE-MRGs observed in macrophages in Result 4. Therefore, C15orf48, UCP2, PPIF, and MGST1 emerge as critical targets for therapeutic intervention in AS.

## 4 Discussion

A substantial body of research has established a close interaction between immune cells and mitochondrial function in AS. Mitochondria not only serve as the primary energy source for organisms but also play critical roles in immune regulation and inflammatory processes (20). Their involvement in immunomodulatory and inflammatory mechanisms during disease progression occurs primarily through three key pathways: (1) Direct involvement in immune cell activation (21, 22). (2) Signaling Regulation (23). (3) Maintenance of Cellular Homeostasis (24). Disruptions in any of these processes can lead to mitochondrial dysfunction, which may contribute to the pathogenesis of related diseases. In conclusion, mitochondria are indispensable for regulating the activation and signaling of immune cells. A comprehensive investigation into the interaction mechanisms between mitochondria and immune cells is crucial for elucidating the molecular pathways involved in immune regulation and for developing therapeutic strategies for AS-related diseases.

Macrophages are a pivotal component of the innate immune system, playing significant roles in the progression of various immune-related diseases through mechanisms such as antigen presentation, polarization, and phagocytosis (25). Recent studies have highlighted the crucial involvement of macrophage differentiation in the initiation, growth, rupture, and healing phases of atherosclerotic plaque formation (26). In AS, macrophages can influence the immune environment by promoting pro-inflammatory M1 phenotypic polarization while reducing anti-inflammatory M2 polarization, thereby exacerbating arterial inflammation and plaque formation (27). Furthermore, research indicates that converting pro-inflammatory macrophages to those with an anti-inflammatory phenotype can inhibit foam cell formation, prevent AS (28). Thus, regulating the transformation between M1 and M2 macrophages presents a promising therapeutic target in AS. In our study, we discovered that mitochondria-related genes are closely linked to macrophage differentiation in AS, as evidenced by transcriptomics combined with single-cell technology analysis. Specifically, our analysis identified that 15 key mitochondrial genes were more enriched in macrophages compared to other cell groups, with C15orf48, UCP2, PPIF, and MGST1 showing alterations in response to macrophage differentiation levels. Importantly, validation using an *in vitro* AS model revealed significant upregulation of C15orf48, UCP2, and PPIF in AS macrophages, while MGST1 expression was notably downregulated. Therefore, we propose that the expression of mitochondria-related genes C15orf48, UCP2, PPIF, and MGST1 may contribute to the development of AS by influencing macrophage differentiation, thereby altering the immune microenvironment in AS.

C15orf48, also known as NMES1, is a mitochondrial protein homologous to the NDUFA4 subunit of cytochrome C oxidase. Recent studies have highlighted its significant upregulation in various inflammatory conditions, including autoimmune, metabolic, cardiovascular, and infectious diseases, which aligns with our findings (29). Although the specific role of C15orf48 in AS remains unexplored, evidence suggests its critical involvement in the inflammatory response as part of the mitochondrial respiratory chain complex IV (30). Clayton (29) et al. have demonstrated that the expression of C15orf48 is a conserved response to inflammatory stimuli, manifesting across multiple inflammation-related pathways. Notably, substantial upregulation of C15orf48 has been observed in conditions such as rheumatoid arthritis and COVID-19, with expression levels correlating with specific macrophage subpopulations (29). Furthermore, additional studies indicate that C15orf48 is overexpressed following macrophage activation and plays a role in modulating inflammatory cytokine expression (31). Given the pivotal role of inflammation in the progression of AS, the elevated expression of C15orf48 observed in our study may contribute to AS development by regulating macrophage-mediated inflammatory responses and influencing immune function.

UCP2 is a pivotal mitochondrial antioxidant protein (32), with emerging evidence suggesting its involvement in AS through the regulation of oxidative stress and immune responses. Moukdar et al. (33) demonstrated that UCP2 expression is significantly elevated in the aorta of atherosclerotic mice. Conversely, UCP2-deficient mice exhibited marked endothelial dysfunction following an atherosclerotic diet, even in the absence of other genetic modifications. Our study corroborates these findings, revealing a significant upregulation of UCP2 expression, which may influence endothelial cell function. However, contrasting evidence exists in the literature, with many studies indicating that UCP2 deficiency exacerbates plaque formation in high-fat diet-induced AS (34). These studies suggest that UCP2 overexpression mitigates immune damage by enhancing mitochondrial function and reducing oxidative stress and inflammation (35). Notably, UCP2 overexpression during inflammatory responses has been shown to modulate M1 macrophage activation by regulating reactive oxygen species (ROS) production, thereby altering immune processes and curbing inflammation (36). Additionally, UCP2 upregulation promotes oxidative phosphorylation, facilitating the reprogramming of M1 macrophages to an M2 phenotype, which further inhibits inflammation in murine models (37). Overall, the role of UCP2 expression in the progression of AS remains contentious, warranting further investigation to elucidate its precise function in this context.

PPIF is a protein-coding gene that encodes a component of the mitochondrial permeability transition pore located in the inner mitochondrial membrane. Similar to C15orf48, research on PPIF in the context of AS is limited. Notably, PPIF has been implicated in the regulation of mitochondrial permeability transition, significantly influencing the early inflammatory response in macrophages (38). Upregulation of PPIF can accelerate the progression of atherosclerotic lesions by promoting macrophage death through the formation of cyclophilin D (CypD), leading to necrotic core formation and increased plaque instability in advanced AS (39). Beyond its effects on macrophages, PPIF directly impacts mitochondrial function, contributing to disease progression. Studies have shown that deletion of PPIF reduces CypD formation, thereby mitigating mitochondrial dysfunction and inflammatory reprogramming (39). Furthermore, increased acetylation of CypD, encoded by PPIF, can exacerbate endothelial dysfunction and hypertension by inducing mitochondrial dysfunction, both of which are critical factors in the pathogenesis of AS (40). In our current study, we observed a significant upregulation of PPIF expression levels, suggesting its involvement in AS development through the aforementioned pathways.

MGST1 is a multifunctional enzyme that facilitates the covalent binding of reduced glutathione (GSH) to hydrophobic and electrophilic substrates, aiding in the degradation of both endogenous and exogenous harmful substances. Recent studies suggest that MGST1 may serve as a potential therapeutic target for AS by modulating the GSH metabolic pathway (41). In our current study, we observed a significant reduction in the expression levels of MGST1 in AS, with MGST1 being linked to various immune responses. However, the specific role of MGST1 within the immune microenvironment of AS remains unclear. It has been demonstrated that MGST1 acts as an inflammatory gene in macrophages, with its expression levels being upregulated by eicosapentaenoic acid (EPA) and docosahexaenoic acid (DHA), potentially contributing to the progression of inflammatory diseases (42). Furthermore, research by Zhang et al. (43) indicated that MGST1 overexpression protects ductal cells from inflammatory injury, while Yang (44) et al. reported that MGST1 overexpression mitigates myocardial ischemia/reperfusion injury by alleviating mitochondrial dysfunction and ferroptosis. Based on these findings, we hypothesize that in the context of AS, reduced MGST1 expression may accelerate disease progression by promoting inflammatory responses.

In conclusion, this study represents a pioneering effort to elucidate the relationship between mitochondria-related genes and the immune microenvironment of AS through comprehensive bioinformatics analysis. Our findings indicate that the mitochondria-related genes C15orf48, UCP2, PPIF, and MGST1 may serve as promising therapeutic targets for future interventions aimed at modulating the AS immune microenvironment. We have substantiated the expression of these four pivotal genes at the cellular level. However, our study is not without limitations. Firstly, the validation of these key genes was confined to the cellular level, and their expression in AS animal models remains to be verified. Secondly, the precise role of mitochondria-related genes in macrophages, along with their specific mechanisms in the progression of AS, remains elusive and warrants further exploration. Lastly, our analysis also highlighted that, beyond macrophages, mitochondria-related genes are significantly enriched in endothelial cells. The interaction between these key genes and AS endothelial cells presents another promising avenue for future research.

## 5 Conclusion

Our study suggests that C15orf48, UCP2, PPIF, and MGST1 may serve as potential therapeutic targets for improving the immune microenvironment in AS. Future in-depth investigations are essential to further elucidate their roles and to identify new therapeutic and prognostic targets for AS and its related complications.

## Data Availability

Publicly available datasets were analyzed in this study. This data can be found here: the Gene Expression Omnibus (GEO) database (GSE159677: https://www.ncbi.nlm.nih.gov/geo/query/acc.cgi?acc=GSE159677; GSE100927: https://www.ncbi.nlm.nih.gov/geo/query/acc.cgi?acc=GSE100927).

## References

[1] Herrington W Lacey B Sherliker P Armitage J Lewington S. Epidemiology of atherosclerosis and the potential to reduce the global burden of atherothrombotic disease. Circ Res. (2016) 4:535–46. 10.1161/CIRCRESAHA.115.307611 26892956

[2] Lin M Hu L Shen S Liu J Liu Y Xu Y Atherosclerosis-related biomarker PABPC1 predicts pan-cancer events. Stroke Vasc Neurol. (2024) 2:108–25. 10.1136/svn-2022-002246 37311641 PMC11103157

[3] Khan AW Paneni F Jandeleit-Dahm KAM. Cell-specific epigenetic changes in atherosclerosis. Clin Sci (Lond). (2021) 9:1165–87. 10.1042/CS20201066 33988232 PMC8314213

[4] Kolaszyńska O Lorkowski J. Symmetry and asymmetry in atherosclerosis. Int J Occup Med Environ Health. (2023) 6:693–703. 10.13075/ijomeh.1896.02171 PMC10743353 37791506

[5] Chen W Li Z Zhao Y Chen Y Huang R. Global and national burden of atherosclerosis from 1990 to 2019: trend analysis based on the global burden of disease study 2019. Chin Med J. (2023) 20:2442–50. 10.1097/CM9.0000000000002839 PMC10586830 37677929

[6] Wolf D Ley K. Immunity and inflammation in atherosclerosis. Circ Res. (2019) 124(2):315–27. 10.1161/CIRCRESAHA.118.313591 30653442 PMC6342482

[7] Wang J Kang Z Liu Y Li Z Liu Y Liu J. Identification of immune cell infiltration and diagnostic biomarkers in unstable atherosclerotic plaques by integrated bioinformatics analysis and machine learning. Front Immunol. (2022) 13:956078. 10.3389/fimmu.2022.956078 36211422 PMC9537477

[8] Zhou Y Wang S Liang X Heger Z Xu M Lu Q Turning hot into cold: immune microenvironment reshaping for atherosclerosis attenuation based on pH-responsive shSiglec-1 delivery system. ACS Nano. (2022) 7:10517–33. 10.1021/acsnano.2c01778 35762565

[9] Vallejo J Cochain C. Heterogeneity of immune cells in human atherosclerosis revealed by scRNA-seq. Cardiovasc Res. (2021) 13:2537–43. 10.1093/cvr/cvab260 34343272 PMC8921647

[10] Ma J Luo J Sun Y Zhao Z. Cytokines associated with immune response in atherosclerosis. Am J Transl Res. (2022) 9:6424–44. PMC9556506 36247305

[11] Ridker PM Everett BM Thuren T MacFadyen JG Chang WH Ballantyne C Antiinflammatory therapy with canakinumab for atherosclerotic disease. N Engl J Med. (2017) 12:1119–31. 10.1056/NEJMoa1707914 28845751

[12] Deguchi JO Aikawa M Tung CH Aikawa E Kim DE Ntziachristos V Inflammation in atherosclerosis: visualizing matrix metalloproteinase action in macrophages *in vivo*. Circulation. (2006) 1:55–62. 10.1161/CIRCULATIONAHA.106.619056 16801460

[13] Zhang Y Miao Y Tan J Chen F Lei P Zhang Q. Identification of mitochondrial related signature associated with immune microenvironment in Alzheimer’s disease. J Transl Med. (2023) 1:458. 10.1186/s12967-023-04254-9 PMC10334674 37434203

[14] Khwaja B Thankam FG Agrawal DK. Mitochondrial DAMPs and altered mitochondrial dynamics in OxLDL burden in atherosclerosis. Mol Cell Biochem. (2021) 4:1915–28. 10.1007/s11010-021-04061-0 33492610

[15] Roy P Orecchioni M. How the immune system shapes atherosclerosis: roles of innate and adaptive immunity. Nat Rev Immunol. (2022) 22(4):251–65. 10.1038/s41577-021-00584-1 34389841 PMC10111155

[16] Zhang C Zhao X Li F Qin J Yang L Yin Q Integrating single-cell and multi-omic approaches reveals Euphorbiae humifusae herba-dependent mitochondrial dysfunction in non-small-cell lung cancer. J Cell Mol Med. (2024) 10:e18317. 10.1111/jcmm.18317 38801409 PMC11129731

[17] Singh A Faccenda D Campanella M. Pharmacological advances in mitochondrial therapy. EBioMedicine. (2021) 65:103244. 10.1016/j.ebiom.2021.103244 33647769 PMC7920826

[18] Ji Y Leng Y Lei S Qiu Z Ming H Zhang Y ZThe mitochondria-targeted antioxidant MitoQ ameliorates myocardial ischemia-reperfusion injury by enhancing PINK1/parkin-mediated mitophagy in type 2 diabetic rats. Cell Stress Chaperones. (2022) 4:353–67. 10.1007/s12192-022-01273-1 PMC9346044 35426609

[19] Rehman MU Sehar N Dar NJ Khan A Arafah A Rashid S Mitochondrial dysfunctions, oxidative stress and neuroinflammation as therapeutic targets for neurodegenerative diseases: an update on current advances and impediments. Neurosci Biobehav Rev. (2023) 144:104961. 10.1016/j.neubiorev.2022.104961 36395982

[20] Casanova A Wevers A Navarro-Ledesma S Pruimboom L. Mitochondria: it is all about energy. Front Physiol. (2023) 14:1114231. 10.3389/fphys.2023.1114231 37179826 PMC10167337

[21] Faas MM de Vos P. Mitochondrial function in immune cells in health and disease. Biochim Biophys Acta Mol Basis Dis. (2020) 10:165845. 10.1016/j.bbadis.2020.165845 32473386

[22] Breda CNS Davanzo GG Basso PJ Saraiva Câmara NO Moraes-Vieira PMM. Mitochondria as central hub of the immune system. Redox Biol. (2019) 26:101255. 10.1016/j.redox.2019.101255 31247505 PMC6598836

[23] Mills EL Kelly B O'Neill LAJ. Mitochondria are the powerhouses of immunity. Nat Immunol. (2017) 5:488–98. 10.1038/ni.3704 28418387

[24] Ma K Chen G Li W Kepp O Zhu Y Chen Q. Mitophagy, mitochondrial homeostasis, and cell fate. Front Cell Dev Biol. (2020) 8:467. 10.3389/fcell.2020.00467 32671064 PMC7326955

[25] Eshghjoo S Kim DM Jayaraman A Sun Y. Macrophage polarization in atherosclerosis. Genes. (2022) 5:756. 10.3390/genes13050756 PMC9142092 35627141

[26] Hou P Fang J Liu Z Shi Y Agostini M. Macrophage polarization and metabolism in atherosclerosis. Cell Death Dis. (2023) 10:691. 10.1038/s41419-023-06206-z PMC10589261 37863894

[27] Jinnouchi H Guo L Sakamoto A Torii S Sato Y Cornelissen A Diversity of macrophage phenotypes and responses in atherosclerosis. Cell Mol Life Sci. (2020) 77(10):1919–32. 10.1007/s00018-019-03371-3 31720740 PMC11104939

[28] Liu X Guo JW Lin XC. Macrophage NFATc3 prevents foam cell formation and atherosclerosis: evidence and mechanisms. Eur Heart J. (2021) 47:4847–61. 10.1093/eurheartj/ehab660 34570211

[29] Clayton SA Daley KK MacDonald L. Inflammation causes remodeling of mitochondrial cytochrome c oxidase mediated by the bifunctional gene C15orf48. Sci Adv. (2021) 50:eabl5182. 10.1126/sciadv.abl5182 34878835 PMC8654286

[30] Li C Tang Y Li Q Liu H Ma X He L The prognostic and immune significance of C15orf48 in pan-cancer and its relationship with proliferation and apoptosis of thyroid carcinoma. Front Immunol. (2023) 14:1131870. 10.3389/fimmu.2023.1131870 36969231 PMC10033576

[31] Xiong M Liu Z. The epithelial C15ORF48/miR-147-NDUFA4 axis is an essential regulator of gut inflammation, energy metabolism, and the microbiome. Proc Natl Acad Sci USA. (2024) 27:e2315944121. 10.1073/pnas.2315944121 PMC11228508 38917002

[32] Tian XY Ma S Tse G Wong WT Huang Y. Uncoupling protein 2 in cardiovascular health and disease. Front Physiol. (2018) 9:1060. 10.3389/fphys.2018.01060 30116205 PMC6082951

[33] Moukdar F Robidoux J Lyght O Pi J Daniel KW Collins S. Reduced antioxidant capacity and diet-induced atherosclerosis in uncoupling protein-2-deficient mice. J Lipid Res. (2009) 50:59–70. 10.1194/jlr.M800273-JLR200 18698091

[34] Luo JY Cheng CK He L Pu Y Zhang Y Lin X. Endothelial UCP2 is a mechanosensitive suppressor of atherosclerosis. Circ Res. (2022) 5:424–41. 10.1161/CIRCRESAHA.122.321187 PMC9390236 35899624

[35] Ding Y Zheng Y Huang J Peng W Chen X Kang X UCP2 ameliorates mitochondrial dysfunction, inflammation, and oxidative stress in lipopolysaccharide-induced acute kidney injury. Int Immunopharmacol. (2019) 71:336–49. 10.1016/j.intimp.2019.03.043 30952098

[36] Yan X Yuan Z Bian Y Jin L Mao Z Lei J Uncoupling protein-2 regulates M1 macrophage infiltration of gingiva with periodontitis. Cent Eur J Immunol. (2020) 1:9–21. 10.5114/ceji.2020.94664 PMC7226558 32425675

[37] Li Y Lv J Liu S Wang Z Gao Y Fan Z Macrophage corpses for immunoregulation and targeted drug delivery in treatment of collagen-induced arthritis mice. Biomaterials. (2025) 314:122867. 10.1016/j.biomaterials.2024.122867 39366181

[38] Priber J Fonai F Jakus PB Racz B Chinopoulos C Tretter L Cyclophilin D disruption attenuates lipopolysaccharide-induced inflammatory response in primary mouse macrophages. Biochem Cell Biol. (2015) 3:241–50. 10.1139/bcb-2014-0120 25728038

[39] Koga JI Umezu R Kondo Y Shirouzu T Orkhonselenge N Ueno H Cyclophilin D induces necrotic core formation by mediating mitochondria-associated macrophage death in advanced atherosclerotic lesions. Atherosclerosis. (2024) 396:118524. 10.1016/j.atherosclerosis.2024.118524 38972156

[40] Dikalova A Fehrenbach D. Mitochondrial CypD acetylation promotes endothelial dysfunction and hypertension. Circ Res. (2024) 11:1451–64. 10.1161/CIRCRESAHA.123.323596 PMC11116043 38639088

[41] Guo B He X. The mechanism of bisphenol S-induced atherosclerosis elucidated based on network toxicology, molecular docking, and machine learning. J Appl Toxicol. (2025):4768. 10.1002/jat.4768 39978769

[42] Allam-Ndoul B Guénard F Barbier O Vohl MC. Effect of n-3 fatty acids on the expression of inflammatory genes in THP-1 macrophages. Lipids Health Dis. (2016) 15:69. 10.1186/s12944-016-0241-4 27044314 PMC4820929

[43] Zhang R Ling X Guo X Ding Z. MGST1 protects pancreatic ductal cells from inflammatory damage in acute pancreatitis by inhibiting ferroptosis: bioinformatics analysis with experimental validation. Int J Mol Sci. (2025) 5:1899. 10.3390/ijms26051899 40076525 PMC11899814

[44] Yang Y Zhao C Li C Lu Z Cao X Wu Q. MGST1 overexpression ameliorates mitochondrial dysfunction and ferroptosis during myocardial ischemia/reperfusion injury after heart transplantation. Int J Biol Macromol. (2025) 299:140135. 10.1016/j.ijbiomac.2025.140135 39848358

